# Symptomatic Treatment of Acute Traveler’s Diarrhea With Acupuncture at Stomach 36 (ST36) and Large Intestine 4 (LI4) Acupuncture Points

**DOI:** 10.7759/cureus.81136

**Published:** 2025-03-25

**Authors:** Lykourgos Christos Alexakis, Angeliki Konstantinou

**Affiliations:** 1 Internal Medicine, Independent Researcher, Frankfurt, DEU; 2 Anesthesiology, St. Katharinen Hospital, Frankfurt, DEU

**Keywords:** acupuncture therapy, acute diarrhea, diarrhea, traveler’s diarrhea, travel medicine, travel-related illness

## Abstract

Traveler’s diarrhea is a common occurrence affecting many travelers visiting tropical countries. Antidiarrheal medications are the mainstream treatment.

We describe a case of typical traveler’s diarrhea in a 44-year-old tourist in Cuba, who was treated with acupuncture in addition to rehydration, without the use of any medication (e.g., antibiotics or anti-motility agents). On the seventh day of the trip, the traveler developed sudden onset, profuse watery diarrhea with mild nausea. During a period of eight hours, the patient passed profuse watery stools five times. Two standard acupuncture needles (0.30 x 30 mm) were inserted by an anesthesiologist trained in acupuncture: one at ST36 (stomach meridian 36) on the left leg and one at LI4 (large intestine meridian 4) on the right hand, for a duration of 30-40 minutes. The exact session was repeated a second time after two days.

After each session, a decrease in the frequency (defecation only once per day) and severity of diarrhea (stool consistency improved as per the Bristol scale) was observed.

Further research on the effectiveness of ST36 and LI4 points acupuncture and acupressure for the symptomatic management of traveler’s diarrhea by means of randomized controlled trials is needed, given the lack of contribution to antimicrobial resistance, the low cost, and the minimal equipment needed for acupuncture and acupressure therapy. If confirmed, such an additional treatment option could be useful in rural, remote, or poor resource settings where the availability of antidiarrheal medications might be limited.

## Introduction

Traveler’s diarrhea is a common occurrence affecting many travelers visiting tropical countries. The exact incidence varies depending on the travel destination, country of origin, season, and trip duration. Attack rates range from 10% to 70% of travelers [1,2]. Although bacteria account for more than 80% of cases, viruses as well as to a lesser extent protozoal parasites may be implicated. Anti-diarrheal medications, such as loperamide, are the mainstream treatment, while antibiotics are reserved for the more severe cases. However, there are concerns about the potential development of antibiotic resistance among bacterial strains [2]. Antimicrobial resistance is a growing global public health threat contributing to a significant number of deaths, especially in low and middle-income countries. Inappropriate antibiotic prescribing is the driving force of this phenomenon [3]. One challenge of the current treatment recommendations for traveler’s diarrhea is the option to use antibiotics in addition to anti-motility agents for moderate diarrhea cases. In these cases, antibiotics might decrease the duration of diarrhea by one to two days if the pathogen is susceptible but there is a risk of becoming colonized by resistant bacterial strains [2].

So far, acupuncture has not been reported in the medical literature as a treatment for traveler’s diarrhea. We report a case of a patient suffering from moderate traveler’s diarrhea who was managed with acupuncture only, in addition to rehydration, which suggests the need for further research in this field.

## Case presentation

A 44-year-old male tourist, originally from Greece, traveled to Cuba for a two-week trip on February 2023. The patient stayed in local rented apartments in Varadero and Havanna. Although he generally followed the hygiene recommendations (“boil it, cook it, peel it, or forget it”), he occasionally ate food from street stalls and systematically washed his teeth with untreated/unfiltered local tap water. His past medical history included ankylosing spondylitis in remission for a decade, vasovagal syncope episodes, migraine, and myopia. The patient was not taking any regular medication.

On the seventh day of the trip, the traveler developed sudden onset, profuse watery diarrhea with mild nausea, which forced him to alter his activity program. No antidiarrheal medications (anti-motility agents) or antibiotics were available among the co-travelers team and the patient was only replenishing fluid and electrolytes by taking oral rehydration solution, drinking black tea, and eating bananas, which contain potassium. One challenge was that the patient denied visiting any local doctor or pharmacy. Also, no laboratory examinations were performed, as per the patient's wishes.

One of the co-travelers, an anesthesiologist trained in acupuncture (240 hours basic course of the German Medical Association for Acupuncture), with six years of practical experience of using acupuncture occasionally in patient care, who is also one of the authors (AK), stepped in and offered to take the medical history, examine the patient, and perform acupuncture for the treatment of diarrhea. The patient agreed.

A physical examination was performed. The patient looked tired but in good general condition. His pulse rate, rhythm, and volume were normal. The abdomen was soft and non-tender, with increased bowel sounds and audible borborygmi. No blood was present in the stools, which were entirely liquid and the patient had no fever (armpit temperature was 37°C). During a period of eight hours (from 8:00 to 16:00), the patient passed five times profuse watery stools, which were type 7 (entirely liquid) according to the Bristol Stool Form Scale [4].

Based on the past and current medical history, the physical examination, and the context, a diagnosis of acute traveler’s diarrhea was made clinically.

The therapeutic intervention consisted of two standard acupuncture needles (0.30 x 30 mm), which were inserted (at around 16:15 time) at two points: one at ST36 (stomach meridian 36, “Zu San Li”) acupuncture point on the left leg and one at LI4 (large intestine meridian 4, “He Gu”) acupuncture point on the right hand [5,6]. The needles were left in place for approximately 30-40 minutes while the patient was relaxed, lying on a bed. The patient was very cooperative and tolerated the acupuncture treatment well. No adverse or unanticipated events were reported.

The ST36 acupuncture point is located three "cun" below the knee and one "cun" lateral to the tibial edge. “Cun” is a traditional Chinese unit of length used in acupuncture. Three “cun” correspond to the width of four fingers. LI4 is located between the 1st and the 2nd metacarpal bones at a point traditionally described as "depressed as a valley” [5,6]. The ST36 acupuncture point was chosen because per current practice, this point is indicated for abdominal complaints, such as gastritis, gastroenteritis, and irritable bowel syndrome. The LI4 acupuncture point was chosen as it is indicated for viral infections [6].

After the acupuncture session, a remarkable improvement was observed. The patient did not defecate again on the first day of illness, although in the past eight hours, he had passed profuse watery stools five times before acupuncture. Audible borborygmi were absent after the acupuncture session and for the rest of the day as confirmed by both the doctor and the patient. The patient also mentioned that he felt intestinal motility decreased significantly during and after the acupuncture session and his abdomen remained calm for the rest of the day.

The next day, the patient had stool only once in the morning, type 6 of the Bristol scale (fluffy pieces with ragged edges), and was able to resume his activities.

On the third day, he had again entirely liquid stools (type 7 Bristol scale) once in the morning. For this reason, a second acupuncture session was performed at the same points and sides with the same duration (30-40 minutes) with the patient lying on a bed. This session was also well tolerated by the patient and no adverse events were reported. Subsequently, the patient had no more stools that day.

On the fourth day, stool consistency was improved to type 5 Bristol scale (soft blobs with clear-cut edges) and the patient defecated only once. Furthermore, the patient had no more diarrhea during the rest of the trip or during the three months of follow-up after his return to his home country (the patient was contacted and asked for diarrhea symptoms).

The patient mentioned that the treatment was very effective with minimal discomfort. He agreed to try acupuncture offered by a co-traveler expatriate doctor because he thought visiting local healthcare services would be more inconvenient. Nevertheless, he did not expect acupuncture to work. For this reason, he was positively impressed with the quick relief of his symptoms following treatment.

## Discussion

Data from animal model studies showed that manual acupuncture and electroacupuncture at the ST36 point might have an anti-inflammatory effect through several mechanisms, including vagus nerve activation or influencing different signaling pathways at the cellular level [7,8]. A systematic review on the use of ST36 point for manual and electroacupuncture as a treatment for sepsis in animal models concluded that it might be effective in reducing sepsis-related organ injuries but also mentioned high heterogeneity and suboptimal methodological quality in the included studies [9].

Concerning the gastrointestinal tract, it was reported that electroacupuncture at the ST36 point can improve different gastrointestinal motility disorders in experimental animals. In a study, this intervention inhibited the accelerated colonic transit, which was induced by restraint stress in rats [10]. In humans, four randomized controlled trials have been performed evaluating manual acupuncture of LI4 and ST36 points for the treatment of infantile colic. Although acupuncture appeared to be effective in alleviating the colic symptoms, the evidence was inconclusive as the sample sizes were small [11]. Specifically in one of these trials, where only LI4 point acupuncture was used, stool frequency data were collected. The stool frequency in the control group remained higher compared to the acupuncture group but was not statistically significant, possibly due to the small sample size. In the same study, almost twice as many parents in the control group remarked that the infant's stools were more watery compared to the acupuncture group [12].

Untreated traveler's diarrhea due to bacteria lasts three to seven days while it lasts two to three days due to viruses. On the other hand, protozoa can cause diarrhea lasting for weeks to months if left untreated [2]. As per the WHO definition, diarrhea is defined as the passage of three or more loose or liquid stools per day (or more frequent passage than is normal for the individual) [13]. As per the current guidelines, the patient in our case report suffered from moderate acute traveler’s diarrhea. This is defined as diarrhea that is distressing or interferes with planned activities. In such moderate cases, loperamide is recommended either as monotherapy or as adjunctive therapy. Additionally, azithromycin or fluoroquinolones may be used [14]. In our patient, after acupuncture at ST36 and LI4 acupuncture points, a decrease in both (1) frequency (after the first session, defecation was decreased to once per day) and (2) severity of diarrhea, as evaluated using the Bristol Stool Form Scale, was observed (after each of the two sessions, stool consistency was improved).

Following a literature search in PubMed, to our knowledge, this is the first report of using acupuncture for the symptomatic management of moderate traveler’s diarrhea. It should be noted that this intervention took place in a trip setting in a tropical country, which is representative of the usual field conditions and target population.

There are many limitations with this study as this is a case report and no causal inferences can be made. The small sample size is prone to selection and measurement bias and without a control group, no statistical significance testing can be done. The observed improvement in our patient could reflect the natural course of the illness. Rehydration, dietary changes, as well as the placebo effect could have contributed to symptom resolution.

On the other hand, patients included in case reports are more diverse and representative of those encountered in routine clinical care and these studies can point to specific scientific areas needing clarification as well as help construct new research questions for further research.

Indeed, further research is needed by means of randomized controlled trials to clarify whether acupuncture and potentially acupressure, which is easier for laypersons to perform, at ST36 and LI4 acupuncture points is an effective intervention for the symptomatic management of mild to moderate traveler’s diarrhea. If confirmed, this method could be useful as an alternative, low-cost, or additional treatment option, which does not contribute to the development of antimicrobial resistance. Such an option would be particularly relevant in rural, remote, or poor resource settings where antidiarrheal medication availability might be limited.

## Conclusions

Following acupuncture at ST36 and LI4 acupuncture points, a decrease in the frequency and severity of diarrhea in a patient suffering from traveler’s diarrhea was observed. Further testing of this finding by means of randomized controlled trials is worthwhile, given the lack of contribution to antimicrobial resistance, the low cost, and the minimal equipment needed for acupuncture and acupressure therapy. Such an option, if confirmed, could be useful in rural, remote, or poor resource settings where antidiarrheal medication availability might be limited.
